# Retraction: NaHS protects against the impairments induced by oxygen-glucose deprivation in different ages of primary hippocampal neurons

**DOI:** 10.3389/fncel.2026.1794007

**Published:** 2026-02-03

**Authors:** Frontiers Editorial Office

The publisher retracts the article cited above.

Following publication, concerns were raised regarding the integrity of the images in the published figures. The authors failed to provide the raw data or a satisfactory explanation during the investigation, which was conducted in accordance with Frontiers' policies. Given the concerns about the validity of the data, and the lack of raw data, the editors no longer have confidence in the findings presented in the article.

This retraction was approved by the Chief Executive Editor of Frontiers. The authors received a communication regarding the retraction and had a chance to respond. This communication has been recorded by the publisher.

Frontiers would like to thank the users on PubPeer for bringing the published article to ourattention.

